# Evolution of Surgical Outcomes in Spinal Metastases from Solid Tumors: A Comparative Analysis over 2 Decades

**DOI:** 10.1055/s-0046-1824730

**Published:** 2026-07-28

**Authors:** Fernando Pacheco dos Santos Ferreira da Silva, Pedro Reggiani Anzuatégui, Ana Valéria Rigolino Teixeira, Fernanda Pinto Garcia, Lucas Emanuel Sauer Larocca, Glauco José Pauka Mello

**Affiliations:** 1 Oncologic Orthopedics Department, Hospital Erasto Gaertner, Curitiba, PR, Brazil; 2 Surgery Department, Health Sciences Sector, Universidade Federal do Paraná (UFPR), Curitiba, PR, Brazil

**Keywords:** mortality, neoplasm metastasis, postoperative complications, spine/surgery, survival, coluna vertebral/cirurgia, complicações pós-operatórias, metástase neoplásica, mortalidade, sobrevida

## Abstract

**Objective:**

To evaluate the evolution of surgical outcomes in patients undergoing surgery for spinal metastases from solid tumors over more than two decades in a single tertiary referral center.

**Methods:**

The present retrospective comparative study included 2 cohorts of patients who underwent surgical treatment for spinal metastases: cohort 1 (2002–2015) and cohort 2 (2018–2024). Overall survival was estimated using the Kaplan-Meier method and compared with the log-rank test. Major postoperative complications (grades III–IV) were analyzed per patient and compared between cohorts.

**Results:**

A total of 301 patients were included (175 in cohort 1 and 126 in cohort 2). The more recent cohort demonstrated significantly improved overall survival (log-rank *p* < 0.001), with a hazard ratio for mortality of 0.61 (95%CI: 0.48–0.78) compared with the earlier cohort. Early survival at 30 and 90 days also improved in cohort 2. Overall complication rates were similar between groups; however, cohort 2 showed a significant reduction in local complications.

**Conclusion:**

Surgical outcomes for spinal metastases improved over time, particularly in early survival and the reduction of local complications. These findings suggest that refinement in patient selection, prognostic stratification, and multidisciplinary perioperative care may have contributed to this temporal improvement.

## Resumo

**Objetivo:**

Avaliar a evolução dos desfechos cirúrgicos em pacientes submetidos à cirurgia para metástases vertebrais de tumores sólidos ao longo de mais de duas décadas em um único serviço terciário.

**Métodos:**

Estudo retrospectivo comparativo envolvendo duas coortes de pacientes submetidos a tratamento cirúrgico para metástases vertebrais: coorte 1 (2002–2015) e coorte 2 (2018–2024). A sobrevida global foi estimada pelo método de Kaplan-Meier e comparada pelo teste log-rank. Complicações maiores pós-operatórias (graus III–IV) foram analisadas por paciente e comparadas entre as coortes.

**Resultados:**

Foram incluídos 301 pacientes (175 na coorte 1 e 126 na coorte 2). A coorte mais recente apresentou aumento significativo da sobrevida global (log-rank *p* < 0,001), com razão de risco para mortalidade de 0,61 (IC95%: 0,48–0,78) em relação à coorte anterior. Observou-se também melhora da sobrevida precoce nos marcos de 30 e 90 dias. A incidência global de complicações foi semelhante entre os grupos, porém houve redução significativa das complicações locais na coorte 2.

**Conclusão:**

Os desfechos cirúrgicos das metástases vertebrais demonstraram melhora ao longo do tempo, especialmente em relação à sobrevida precoce e à redução das complicações locais. Esses achados sugerem que o refinamento da seleção de pacientes, da estratificação prognóstica e do cuidado perioperatório multidisciplinar pode ter contribuído para essa evolução.

## Introduction

Spinal metastases are a frequent manifestation of the spread of solid tumors and represent a relevant cause of pain, mechanical instability, and neurological deficit, with a direct impact on autonomy and quality of life. 1 Given this scenario, the indication for surgical treatment should balance potential functional benefit with perioperative risk, especially in patients with advanced systemic disease and clinical fragility. 2

Surgery can provide important clinical benefits, such as neural decompression, stabilization of the spine, and relief of refractory pain, especially in scenarios of spinal cord instability and compression. 3 However, these are potentially extensive interventions associated with considerable morbidity, particularly in frail patients with limited life expectancy. In this context, the decision between surgical and non-surgical approaches—such as radiotherapy, systemic therapies, and palliative care—requires careful selection of candidates and integration of treatment into a global oncology strategy. 4 Still, contemporary evidence suggests that appropriately selected patients may show clinically relevant improvement in health-related quality of life after surgery for spinal metastases, reinforcing the importance of an individualized indication. 1 5

Among the outcomes used in decision-making, early survival after surgery—especially at the 30- and 90-day milestones—plays a central role. 5 6 Early deaths tend to indicate a disproportion between the aggressiveness of the procedure and the expected benefit, which is why thresholds such as 90 days are often used as a pragmatic reference to weigh risk, benefit, and therapeutic proportionality. 7 8

Several prognostic stratification systems have been proposed to assist surgical decision-making in patients with spinal metastases. Among them, we highlight classical scores based predominantly on tumor biology and disease extent, such as those of Tomita and Tokuhashi, as well as models that incorporate additional clinical variables, including functional status and comorbidities. In the national context, simple tools for assessing frailty have also been described, such as the three-predictor model by Anzuatégui et al., based on readily obtainable clinical and laboratory variables. 9 10 11 Finally, machine-learning-based predictive models have been developed with potentially superior discriminative performance, but at the expense of greater complexity and less immediate bedside applicability. 12 13

Despite advances in the use of models and scores, relevant gaps remain in the literature, particularly regarding the temporal evolution of surgical outcomes within the same service and amid transitions in care practices, technologies, and resources. In addition, historical series often exhibit heterogeneity in indications, techniques, and outcome recording, especially given the inherent limitations of retrospective data collection.

Therefore, the present study aimed to evaluate the evolution of surgical outcomes in patients with spinal metastases from solid tumors over more than two decades in a single service, with emphasis on early and overall survival, the occurrence of complications, and to discuss factors potentially associated with this evolution.

## Methods

The present is a retrospective, comparative, and longitudinal study conducted in a single service that evaluated patients undergoing surgical treatment for spinal metastases secondary to solid tumors. Two distinct cohorts were analyzed: cohort 1, a retrospective cohort of patients treated between 2002 and 2015, and cohort 2, a prospective cohort of patients treated between 2018 and 2024. Despite differences in their origins, both cohorts were evaluated retrospectively in the present study using previously recorded clinical and surgical data.

Regarding the surgical technique, in both cohorts, the treatment consisted of neural decompression with instrumented stabilization to maintain stability and mechanical control. Over the period studied, there was a progressive evolution of implants and instruments, with improvements in material quality and the availability of fixation and reconstruction resources, reflecting the modernization of the service's surgical arsenal, without changing the fundamental objectives of the procedure. The characterization of the surgical level and approach was recorded for comparative analysis between the cohorts.

The primary objective was to compare surgical outcomes over more than 2 decades of evolution within the same service, with an emphasis on overall survival, survival at 30, 90, and 365 days, and the incidence of postoperative complications. As secondary objectives, we sought to determine the incidence of local and systemic complications, characterize their severity, and identify factors associated with the differences observed between the cohorts.

Categorical variables were described by absolute frequencies and percentages, while survival outcomes were reported as medians with corresponding 95%CIs. Categorical variables were compared between cohorts using the Chi-squared or Fisher's exact test, depending on the expected frequencies. Overall survival was estimated using the Kaplan-Meier method, defined as the time from the surgical procedure to death. Patients with no death record until the last contact, including those with loss of follow-up, were considered censored on the date of the last available record. The comparison between the survival curves of the cohorts was performed using the log-rank test. Additionally, the Cox proportional hazards model was used to estimate hazard ratios (HRs) and their 95%CIs.

The analysis of postoperative complications was conducted according to two distinct approaches. For the purposes of the present study, only major complications (grades III–IV) were considered clinically relevant, according to the classification of Rampersaud et al. 14 Initially, an analysis was performed per patient, considering the occurrence of at least one local or systemic complication during follow-up. In a complementary way, we proceeded with event-level analysis, allowing the same patient to contribute more than one complication, and this approach was presented exclusively descriptively. The comparison between proportions was performed using the Chi-squared test, and Fisher's exact test was used when expected frequencies were fewer than 5. When relevant, HRs were estimated with their 95% CIs. Inferential analysis was restricted to variables with sufficient events to apply statistical tests; the others were presented descriptively.

All statistical tests were 2-tailed, adopting a significance level of *p* < 0.05. Statistical analyses were performed using MedCalc Statistical software (MedCalc Software Ltd.), version 17.6.

The study was approved by the Research Ethics Committee under CAAE number: 88280725.1.0000.0098, opinion No. 7.556.572, with waiver of the Informed Consent Form.

## Results

**Fig. 1:**
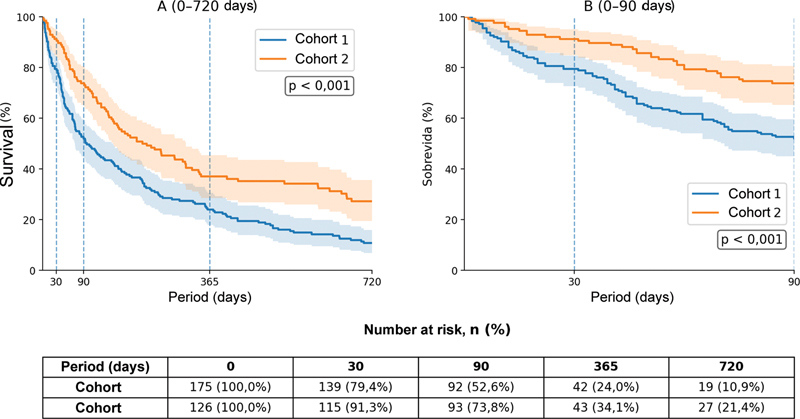
Kaplan-Meier curves of overall survival comparing cohorts 1 and 2. ( **A** ) Analysis of the period of 0 to 720 days; ( **B** ) extension of the initial period (0–90 days). Shaded areas correspond to 95% confidence intervals. The lower table shows the number of patients at risk at each time point, expressed in absolute numbers and percentage (n %]).

The demographic and clinical characteristics of the cohorts are presented in Tables 1 2 . Regarding the outcomes, there was a difference in overall survival between the cohorts, as illustrated in Fig. 1 . The numerical parameters for overall survival, their statistical comparisons, and synthesis at the 30-, 90-, and 365-day milestones are described in Table 3 .

**Table 1:** Sample characteristics

Variable	Cohort 1, n (%)	Cohort 2, n (%)	Total, n (%)
**Number of patients**	175	126	301
**Sex**			
Male	96 (54.9)	58 (46.0)	154 (51.2)
**Age, years**			
Mean	59.1	58.1	—
Median	60.0	58.0	—
**Level operated**			
Cervical/Cervicothoracic	9 (5.1)	8 (6.3)	17 (5.6)
Thoracic	57 (32.6)	53 (42.1)	110 (36.5)
Thoracolumbar	63 (36.0)	49 (38.9)	112 (37.2)
Lumbar/Lumbosacral	42 (24.0)	16 (12.7)	58 (19.3)
Multiple	4 (2.3)	0 (0)	4 (1.3)
**Surgical approach**			
Anterior	3 (1.7)	0 (0)	3 (1.0)
Posterior	172 (98.3)	126 (100)	298 (99.0)

**Note:** Data expressed in absolute number (n) and percentage (%), when applicable.

**Table 2:** Histological types by cohort

Histological type	Cohort 1, n (%)	Cohort 2, n (%)	Total, n (%)	*p*
**Prostate**	51 (29.1)	21 (16.7)	72 (23.9)	0.014
**Breast**	43 (24.6)	37 (29.4)	80 (26.6)	0.358
**Unknown**	20 (11.4)	3 (2.4)	23 (7.6)	0.004
**Lung**	11 (6.3)	12 (9.5)	23 (7.6)	0.380
**Cervix**	12 (6.9)	8 (6.3)	20 (6.6)	1.000
**Head and neck**	8 (4.6)	7 (5.6)	15 (5.0)	0.791
**Kidney**	7 (4.0)	10 (7.9)	17 (5.6)	0.205
**Digestive tract**	6 (3.4)	14 (11.1)	20 (6.6)	0.010
**Melanoma**	6 (3.4)	3 (2.4)	9 (3.0)	0.739
**Other**	6 (3.4)	4 (3.2)	10 (3.3)	1.000
**Sarcoma**	2 (1.1)	4 (3.2)	6 (2.0)	0.241
**Undefined**	2 (1.1)	2 (1.6)	4 (1.3)	1.000
**Thyroid**	1 (0.6)	1 (0.8)	2 (0.7)	1.000

**Notes:** The category “Unknown” refers to tumors of an unidentified primary site, clinically characterized by rapid disease progression. The “Other” category includes, in cohort 1, right upper limb PNET (n = 1), ovarian tumors (n = 2), testicular tumor (n = 1), adrenal tumor (n = 1), and germinal tumor (n = 1); and, in cohort 2, paraganglionic tumor (n = 1), liver tumor (n = 2), and bile duct tumor (n = 1).

**Table 3:** Overview of survival in cohorts

Variable	Cohort 1	Cohort 2	*p*
**Survival ≥ 30 days, n (%)**	139 (79.4)	115 (91.3)	0.005
**Survival ≥ 90 days, n (%)**	92 (52.6)	93 (73.8)	< 0.001
**Survival ≥ 365 days, n (%)**	42 (24.0)	42 (33.3)	0.075
**Overall survival: median in days (95%CI)**	97 (71–154)	228 (154–313)	< 0.001
**Hazard ratio – cohort 2 versus cohort 1 (95%CI)**	—	0.61 (0.47–0.78)	< 0.001

**Notes:** Estimated hazard ratio considering cohort 1 as reference.

**Fig. 2:**
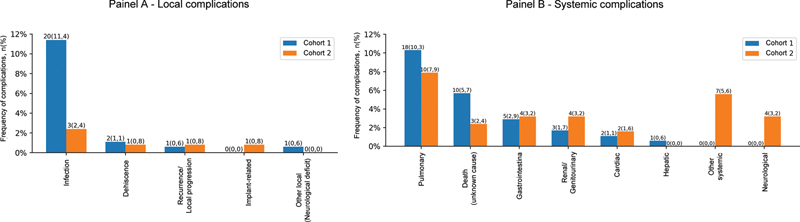
Comparison of local and systemic complications between cohorts. ( **A** ) Local complications; ( **B** ) systemic complications. Values expressed as absolute number (N) and percentage (%), calculated on the total number of patients in each cohort. Some categories occurred exclusively in one of the cohorts.

Regarding complications, the overall incidence per patient and per event is shown in Table 4 . The comparison between the cohorts showed a statistically significant difference only for local complications. The descriptive details of local and systemic complications are illustrated in Fig. 2 and Tables 5 6 .

**Table 4:** Overview of complications in cohorts

Variable	Cohort 1, n (%)	Cohort 2, n (%)	*p*
**A. Patients with complications**			
Any complication	60 (34.3)	34 (27.0)	0.208
≥ 1 local complication	23 (13.1)	6 (4.8)	0.017
≥ 1systemic complication	37 (21.1)	28 (22.2)	0.887
No complication	115 (65.7)	92 (73.0)	0.208
**B. Complication by event**			
Local complication	23	6	—
Systemic complication	39	34	—
Total complication events	62	40	—

**Notes:** The number of patients with complications refers to individuals who experienced at least one adverse event during follow-up. The number of complications per event corresponds to the total number of events recorded, and more than one complication per patient may occur. Percentages and calculation of p are not presented for the number of complications by events, as these represent absolute frequencies and do not constitute independent observations.

**Table 5:** Description of local and systemic complications of cohort 1

Type of complication	n (%)	Subtype	n (%)
**Local complications**			
Infection	20 (11.4)	Surgical wound infection	17 (9.7)
		Surgical wound infection + dehiscence	2 (1.1)
		Infected hematoma	1 (0.6)
Dehiscence	2 (1.1)	Surgical wound dehiscence	2 (1.1)
Other	1 (0.6)	Paralysis on postoperative day 1 (local neurological deficit)	1 (0.6)
**Systemic complications**			
Pulmonary	18 (10.3)	Pneumonia	14 (8.0)
		Noninfectious respiratory failure	3 (1.7)
		Pulmonary thromboembolism	1 (0.6)
Death (unknown cause)	10 (5.7)	—	10 (5.7)
Gastrointestinal	5 (2.9)	Upper gastrointestinal bleeding	3 (1.7)
		Lower gastrointestinal bleeding	2 (1.1)
Renal/Genitourinary	3 (1.7)	Acute kidney injury	2 (1.1)
		Genitourinary tract infection	1 (0.6)
Cardiac	2 (1.1)	Congestive heart failure (with death)	1 (0.6)
		Ischemic cardiac complication	1 (0.6)
Hepatic	1 (0.6)	Acute liver failure	1 (0.6)

**Notes:** Values expressed as absolute number (N) and percentage (%) calculated on the total patients in the cohort. “—” indicates absence of applicable subtype.

**Table 6:** Description of local and systemic complications of cohort 2

Type of complication	n (%)	Subtype	n (%)
**Local complications**			
Infection	3 (2.4)	Surgical wound infection	3 (2.4)
Dehiscence	1 (0.8)	Surgical wound dehiscence with new suture	1 (0.8)
Recurrence	1 (0.8)	Local tumor progression with early paraplegia	1 (0.8)
Implant-related	1 (0.8)	Screw loosening/reoperation	1 (0.8)
**Systemic complications**			
Pulmonary	10 (7.9)	Pneumonia	7 (5.6)
		Noninfectious respiratory failure	2 (1.6)
		Pleural effusion	1 (0.8)
Other systemic	7 (5.6)	Systemic metabolic/hematologic disorder	3 (2.4)
		Soft tissue infection	2 (1.6)
		Systemic viral infection (coronavirus disease 2019)	1 (0.8)
		Sepsis with no defined focus	1 (0.8)
Neurological	4 (3.2)	Central nervous system–tumor–related seizures	3 (2.4)
		Neurological symptoms due to brain metastasis	1 (0.8)
Gastrointestinal	4 (3.2)	Obstructive acute abdomen	1 (0.8)
		**Upper gastrointestinal bleeding**	1 (0.8)
		**Lower gastrointestinal bleeding**	1 (0.8)
		Acute pancreatitis	1 (0.8)
Renal/Genitourinary	4 (3.2)	Genitourinary tract infection	3 (2.4)
		Acute kidney injury	1 (0.8)
Death (unknown cause)	3 (2.4)	—	3 (2.4)
Cardiac	2 (1.6)	Intraoperative cardiorespiratory arrest (with death)	1 (0.8)
		Infective endocarditis	1 (0.8)

**Notes** : Values expressed as absolute number (N) and percentage (%) calculated on the total patients in the cohort. “—” indicates absence of applicable subtype.

## Discussion

The present study demonstrated a significant improvement in surgical outcomes over more than 2 decades within the same service, with an emphasis on increasing overall survival and, more importantly, improving early survival at 30 and 90 days, as well as reducing local complications. These findings suggest that the observed evolution is not limited to isolated technical advances but reflects a broader transformation in the approach to patients with spinal metastases, involving more careful selection, maturation of surgical decision-making, and greater multidisciplinary integration of care. 2 3

Early survival after surgical treatment is an outcome of great clinical relevance in the context of spinal metastases. Patients who die in the first 30 to 90 days after surgery, in general, have limited clinical benefit with extensive interventions, which reinforces the importance of an accurate and individualized surgical indication. Previous studies have shown that early mortality is strongly associated with systemic prognostic factors, tumor burden, and functional reserve, and should be considered centrally in the therapeutic decision. In this scenario, the improvement observed in these temporal milestones between the cohorts suggests a substantial advance in the ability to more accurately identify the patients most likely to benefit from surgical treatment. 4 5 6

The evolution of prognostic stratification emerges as one of the main factors associated with the improvement in early survival observed in this study. Traditionally, classical scoring systems have represented key milestones in the attempt to systematize surgical indications in patients with spinal metastases. With advances in systemic therapies and perioperative support, evidence suggests that some of these scores may underestimate life expectancy in specific subgroups, motivating contemporary comparisons among multiple models and the development of more refined tools that incorporate better-performing clinical, laboratory, and oncological variables to predict early and overall survival. 7 8 9 10 11

In this context, contemporary predictive models and prospective validations have demonstrated moderate-to-good discriminative capacity for clinically relevant outcomes, including early mortality, thereby reinforcing the evolution of surgical decision-making toward reducing potentially futile interventions and maximizing individual benefit. 12 13 15 16 17 Along these lines, the predictive model proposed by Anzuatégui et al., 11 developed in a national population, represents an important contribution to the Brazilian context. In addition, a recent prospective external validation of this 3-predictor frailty model has demonstrated acceptable performance in predicting 90-day survival, reinforcing its usefulness as a simple decision-support tool across diverse clinical scenarios. 18

In addition to the more appropriate patient selection, the improvement in surgical outcomes observed over time can also be attributed to the evolution of perioperative care and surgical techniques. The consolidation of multidisciplinary approaches, integrating orthopedic oncology surgeons, clinical oncologists, radiotherapists, anesthesiologists, intensive care and hospital medicine teams, as well as stomatherapy professionals, enabled more structured therapeutic planning and more efficient postoperative management, with the potential to standardize the management and care of surgical wounds. This model of integrated care has been widely recognized as essential in the contemporary treatment of spinal metastases, contributing to reduced complications and better symptomatic control. 19 20

The reduction in local complications observed in the most recent cohort reinforces this concept. Surgical complications, especially surgical site infections, represent a critical factor in the risk-benefit balance of surgery in cancer patients, being associated with worse functional prognosis and survival. The progressive adoption of preventive measures, such as stricter perioperative protocols, optimization of clinical control, and greater technical standardization, probably played a relevant role in this outcome. 14 20

In addition, in recent years, the use of topical intralesional vancomycin has become part of the routine of care as an adjuvant measure to prevent surgical site infection. Although the studies show divergent results regarding the effectiveness of this strategy, it is possible that its adoption over time contributed, at least in part, to the lower infection frequency observed in the most recent cohort, alongside other changes related to technical evolution and perioperative care. 21 22

Another relevant aspect of the current study lies in the detailed description of complications associated with surgery for spinal metastases. While much of the literature addresses these events in aggregate or is limited to binary outcomes, such as the presence or absence of major complications, the detailed characterization presented in Table 5 allows a more faithful understanding of perioperative morbidity in this population. Previous studies have already highlighted the limitations inherent in capturing and classifying adverse events in spine surgery, especially in cancer patients, in which systemic complications may reflect both the impact of the surgical procedure and the evolution of the underlying disease and its comorbidities. 14 23

The changes observed in the histological profile over time also deserve consideration. The reduction in the proportion of cases with unknown primary origin suggests improved diagnostic methods and greater integration of patients into global cancer care. In the present study, a relative increase in metastases of digestive tract origin and a reduction in cases of prostate origin were observed, findings that may reflect both epidemiological changes and the greater effectiveness of systemic treatments and radiotherapy in specific subgroups, with implications for the selection of patients referred for surgery. 24 25 26 27 28

From a scientific perspective, the current study is unprecedented in the national context, offering a robust, comparative analysis of the evolution of surgical treatment for spinal metastases in the Brazilian population over two decades within a single service. The scarcity of large national series with this scope limits the extrapolation of international data to the local reality, making the findings presented here particularly relevant to clinical practice in Brazil.

Despite its strengths, the study has limitations that should be recognized. The retrospective nature of the analysis may introduce biases inherent to data collection and information completeness, especially in older periods, when physical records and changes in care teams may have impacted the standardization of records. In addition, the absence of systematized functional data and the potential bias in surgical indications over time constitute further limitations. Still, such weaknesses reflect actual clinical practice and do not invalidate observed trends.

In summary, the results of the present study indicate that improvements in early survival and reductions in local complications over time are strongly associated with the refinement of patient selection, the maturation of prognosis-based surgical decision-making, and the evolution of multidisciplinary care. These findings reinforce the role of surgery as part of an individualized and integrated therapeutic strategy aimed at maximizing clinical benefit and minimizing morbidity in patients with spinal metastases. The summary of the main points of this section is highlighted in Table 7 .

**Table 7:** Interpretative summary of differences between cohorts

Domain	Interpretive rationale
**Early survival**	Improved overall and early survival at 30 and 90 days in Cohort 2.
**Local complications**	The more recent cohort showed a lower frequency of local complications after surgical treatment of spinal metastases.
**Prognostic stratification**	Emphasis on the use of prognostic stratification for surgical indication in spinal metastases in the more recent period.
**Perioperative care**	Greater structuring of multidisciplinary perioperative care over time, particularly postoperative clinical support.

## Conclusion

Over more than 2 decades, there has been a progressive improvement in surgical outcomes among patients with spinal metastases treated within a single service, with an emphasis on increasing early survival and reducing local complications. These findings reinforce the importance of refining patient selection and prognostic stratification, advancing technology, and implementing multidisciplinary perioperative care to optimize outcomes. Thus, surgery should be individualized and carefully indicated for patients with a greater likelihood of clinical benefit to maximize gains and minimize treatment-associated morbidity.
